# Inducible Siphoviruses in superficial and deep tissue isolates of *Propionibacterium acnes*

**DOI:** 10.1186/1471-2180-8-139

**Published:** 2008-08-15

**Authors:** Rolf Lood, Matthias Mörgelin, Anna Holmberg, Magnus Rasmussen, Mattias Collin

**Affiliations:** 1 Department of Clinical Sciences, Division of Infection Medicine, BMC-B14, Lund University, SE-221 84 Lund, Sweden

## Abstract

**Background:**

*Propionibacterium acnes *is a commensal of human skin but is also known to be involved in certain diseases, such as acne vulgaris and infections of orthopaedic implants. Treatment of these conditions is complicated by increased resistance to antibiotics and/or biofilm formation of *P. acnes *bacteria. *P. acnes *can be infected by bacteriophages, but until recently little has been known about these viruses. The aim of this study was to identify and characterize inducible phages from *P. acnes *on a genetic and morphological basis.

**Results:**

More than 70% (65/92) of *P. acnes *isolates investigated have inducible phages, classified morphologically as Siphoviruses. The phages have a head of 55 nm in diameter and a tail of 145–155 nm in length and 9–10 nm in width. There was no difference in carriage rate of phages between *P. acnes *isolates from deep infections and isolates from skin. However, there was a significant lower carriage rate of phages in *P. acnes *biotype IB, mostly attributed to the low carriage rate of inducible phages in biotype IB isolated from deep tissue. Most phages have a strong lytic activity against all *P. acnes *isolates with inducible phages, but have less lytic activity against isolates that have no prophages. Phages only infected and lysed *P. acnes *and not other closely related propionibacteria. All phages could infect and lyse their non-induced parental host, indicating that these prophages do not confer superinfection immunity. The phages have identical protein pattern as observed on SDS-PAGE. Finally, sequencing of two phage genes encoding a putative major head protein and an amidase and showed that the phages could be divided into different groups on a genetic basis.

**Conclusion:**

Our findings indicate that temperate phages are common in *P. acnes*, and that they are a genetically and functionally homogeneous group of Siphoviruses. The phages are specific for *P. acnes *and do not seem to confer superinfection immunity.

## Background

*Propionibacterium acnes *is regarded as a commensal of human skin, but is also known to be involved in different infections such as acne vulgaris [1] and infections with orthopaedic implants [2,3]. Treatment of acne vulgaris is complicated by bacterial resistance to commonly used antibiotics [4-6]. *P. acnes *infections of joint prostheses are probably much more common than previously thought [7], and treatment is complicated by biofilm formation on the foreign material [8]. The complete genome of a *P. acnes *isolate was recently sequenced by Brüggemann *et al *2004 [9], showing that *P. acnes *possesses several putative virulence genes including hemolysins and co-hemolysins (CAMP factors) [10-12]. The sequenced genome only contains one cryptic prophage, and in general there is limited knowledge about phages from *P. acnes*.

Bacteriophages can enter two principally different life cycles, lytic or lysogenic. In the lytic life cycle a bacteriophage attaches to the bacterial cell and injects its genetic material. This genetic material is directly replicated, early and late phage genes are transcribed, proteins are translated, new phage particles are formed, and the bacterium is ultimately lysed to release the progeny. In the lysogenic life cycle, most known phages integrate their genome into the host genome by specific attachment and recombination events. There have been reports about phages existing as extrachromosomal circular or linear plasmid prophages, as a part of their lysogenic cycle [13-15]. These so called prophages become integrated parts of the genome and are replicated together with the bacterial genome during cell division. Prophages account for much of the genetic diversity seen in bacteria and often carry genes that are beneficial for the bacteria, including toxins and other virulence factors [16-19].

Almost 30 years ago, it was reported that 18% of *P. acnes *isolates are carriers of bacteriophages [20], but little is known about these phages and their potential impact on virulence. Studies of *P. acnes *phages have been undertaken to establish a phage typing system to distinguish between different types of *P. acnes *[21,22] as an alternative to use fermentative and serological methods [23]. Many studies on phages have been done on other propionibacteria as *Propionibacterium freudenreichii *mainly due to the research impact for dairy industry [24-28].

Recently Farrar *et al *sequenced the first genome of a *P. acnes *lytic phage [29]. The phage was classified as a Siphovirus with a genome of 29,739 bp encoding 48 putative genes. Characterization of phages from *P. acnes *gives a deeper understanding of the relationship between phages and bacteria, and may eventually lead to a new therapy to treat *P. acnes *infections. In this study, we have induced, isolated, and characterized 65 temperate bacteriophages from different *P. acnes *isolates. The phages are all classified as Siphoviruses and can be divided into different groups based on dissimilarities in two genes encoding a putative major head protein and an amidase.

## Results

### Carriage of phages

**Figure 1:**
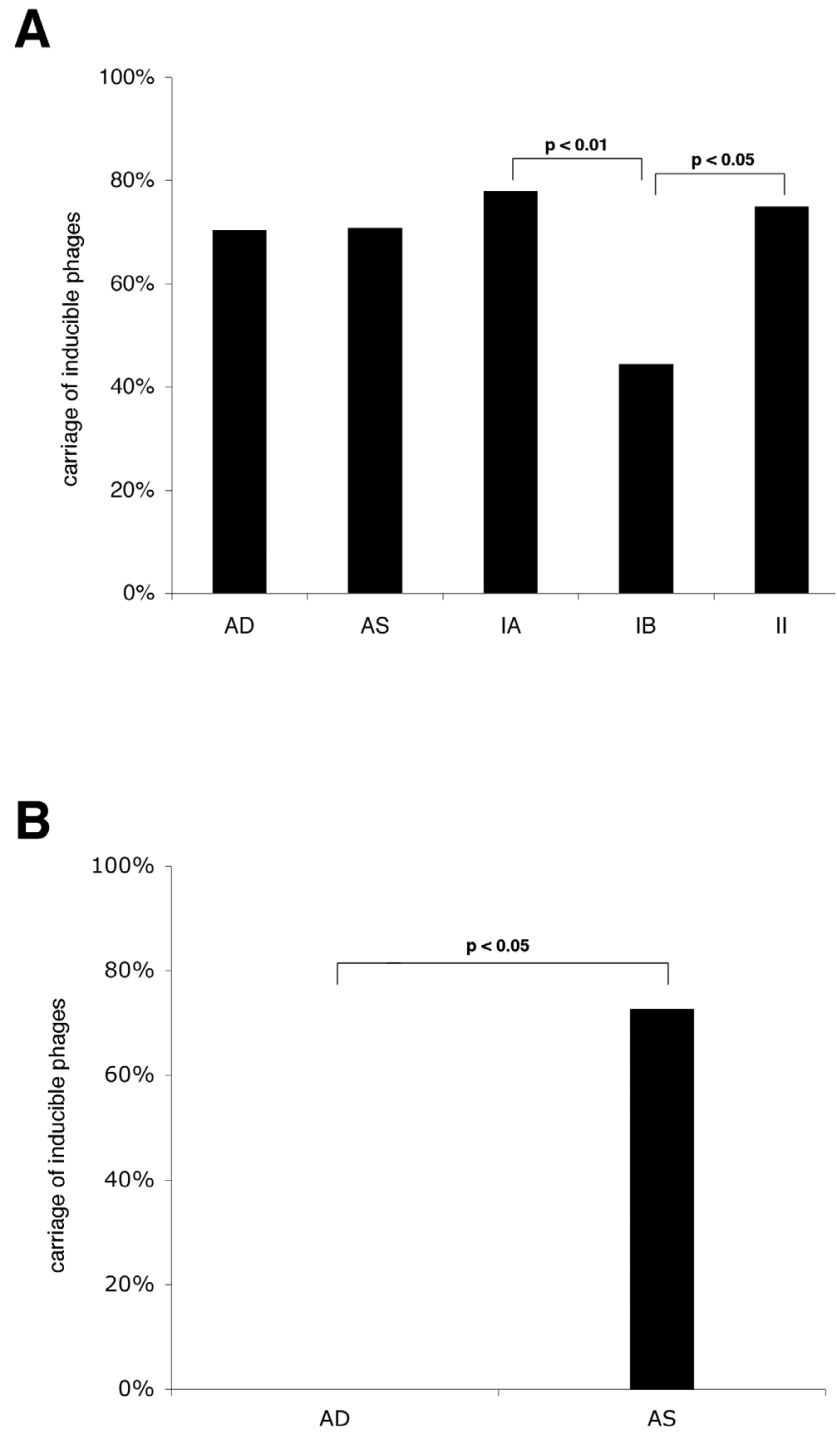
**Carriage rate of phages in different groups and biotypes of *P. acnes***. Phages were induced with 2 μg/ml mitomycin C, lysate sterile filtered and stored for seven days to screen out unstable phages. The lysate was then applied at different concentrations to an overlay plate with the host isolate. If plaques were observed after two days, the sample was regarded positive for phages. (A) A comparison in carriage rate of inducible phages between deep isolates (AD), skin isolates (AS) and biotype IA, IB and II. (B) A comparison of carriage rate of inducible phages in biotype IB between deep isolates and isolates from skin induced by 2 μg/ml mitomycin C.

Since not much is known about the presence and carriage rate of bacteriophages in *P. acnes*, we investigated this in relation to both the site of isolation (superficial or deep infections) and to biotype. To investigate if *P. acnes *had prophages that could be induced to enter the lytic life cycle, we stimulated 92 different *P. acnes *isolates (see table 1) with 2 μg/ml mitomycin C to induce prophages, followed by analysis of the plaque forming capacity of lysates on the noninduced parental isolates. Plaques were clear with well-defined edges and had a diameter of 6–7 mm. Bacteriophages could be induced in more than 70% of the isolates examined. In this study, we have used different isolates of *P. acnes *(Holmberg *et al*, unpublished) from deep tissue (AD-isolates, mainly isolated from infections of foreign material as hip prosthesis and sternal wires), and from the skin (AS-isolates, from the skin of healthy individuals). AD-isolates and AS-isolates had a carriage-rate of 70.5% respectively 70.8% (see Figure 1A).

**Table 1:** *P. acnes *isolates used, result of biotyping and obtained phage isolates.

Isolate	Biotype	Phage	Major Head
AD1	IA	-	-
AD2	IA	PAD2*	EU302613
AD3	IB	-	-
AD4	IA	PAD4*	EU302614
AD5	IA	PAD5*	EU302615
AD6	IA	PAD6*	EU302616
AD7	II	PAD7*	EU302617
AD8	IA	PAD8*	EU302618
AD9	IA	PAD9*	EU302619
AD10	II	PAD10*	EU302620
AD11	IA	PAD11*	EU302621
AD12	IB	-	-
AD13	II	PAD13*	EU302622
AD14	II	PAD14*	EU302623
AD15	IA	-	-
AD16	II	-	-
AD17	IA	PAD17*	EU302624
AD18	II	-	-
AD19	IA	PAD19*	EU302625
AD20	II	PAD20*	EU302626
AD21	II	PAD21*	EU302627
AD22	IA	PAD22*	EU302628
AD23	IA	PAD23*	EU302629
AD24	II	PAD24*	EU302630
AD25	IA	PAD25*	EU302631
AD26	IB	-	-
AD27	IB	-	-
AD28	IA	PAD28*	EU302632
AD29	IA	-	-
AD30	IA	PAD30*	EU302633
AD31	II	-	-
AD33	IB	-	-
AD35	IA	PAD35	EU302634
AD36	IA	PAD36*	EU302635
AD38	IA	PAD38	EU302636
AD39	IB	-	-
AD40	IA	PAD40*	EU302637
AD41	IA	PAD41*	EU302638
AD42	IA	PAD42*	EU302639
AD43	IB	-	-
AD44	IA	PAD44	EU302640
AD45	IA	PAD45	EU302641
AD47	IA	PAD47	EU302642
AD48	IA	PAD48	EU302643
AS1	IB	-	-
AS2	IA	PAS2	EU302610
AS3	IB	PAS3	EU302611
AS4	IA	PAS4	EU302651
AS5	IA	-	-
AS6	IA	PAS6	EU302612
AS7	IA	PAS7	EU302652
AS8	II	PAS8	EU302653
AS9	II	PAS9	EU302654
AS10	IA	PAS10	EU302655
AS11	IB	PAS11	EU302656
AS12	II	PAS12	EU302657
AS13	IB	PAS13	EU302658
AS14	IA	-	-
AS16	IB	PAS16	EU302659
AS18	IB	-	-
AS20	II	-	-
AS21	II	-	-
AS22	II	PAS22	EU302660
AS23	IB	PAS23	EU302661
AS24	IA	PAS24	EU302662
AS25	IA	PAS25	EU302663
AS26	IA	PAS26	EU302664
AS27	IA	PAS27	EU302665
AS28	II	-	-
AS29	II	PAS29	EU302666
AS30	IA	PAS30	EU302667
AS31	IA	PAS31	EU302668
AS32	IA	-	-
AS33	IA	-	-
AS34	IA	-	-
AS35	IA	PAS35	EU302669
AS37	IB	PAS37	EU302670
AS38	IB	-	-
AS39	II	PAS39	EU302671
AS40	II	PAS40	EU302644
AS41	II	PAS41	EU302645
AS42	II	PAS42	EU302646
AS43	II	PAS43	EU302647
AS44	IA	PAS44	EU302607
AS45	IA	-	-
AS46	IB	PAS46	EU302608
AS47	IA	PAS47*	EU302648
AS48	IA	-	-
AS49	IB	PAS49	EU302649
AS50	IA	PAS50*	EU302609
AS51	IA	-	-
AS52	II	PAS52	EU302650

* phages from which high quality TEM micrographs were obtained.

Bacterial isolates that did not have any inducible phages using mitomycin C were screened for prophages using a PCR-based approach by amplification of the gene encoding a putative major head protein and *recA *as a positive control. Only one of the isolates, AS14, was positive in the major head PCR, indicating that only 1/9 of these isolates has phages with similarities to the known major head gene (data not shown).

The carriage rate of inducible temperate phages in the different biotypes was also examined. Biotypes had previously been determined by sequencing of *recA *(Holmberg *et al*, unpublished). Biotype IA had a higher carriage rate than IB (p < 0.01), as did biotype II (p < 0.05). Since biotype IB had a lower carriage rate compared to the other two biotypes, we compared the carriage rate of phages in the isolates determined as biotype IB between deep and superficial isolates of *P. acnes*. The carriage rate in biotype IB was significantly higher in superficial isolates as compared with isolates from deep infections, since none of the deep tissue isolates biotyped as IB had inducible phages (p < 0.05, see Figure 1B).

### Bacteriophage morphology and classification

**Figure 2:**
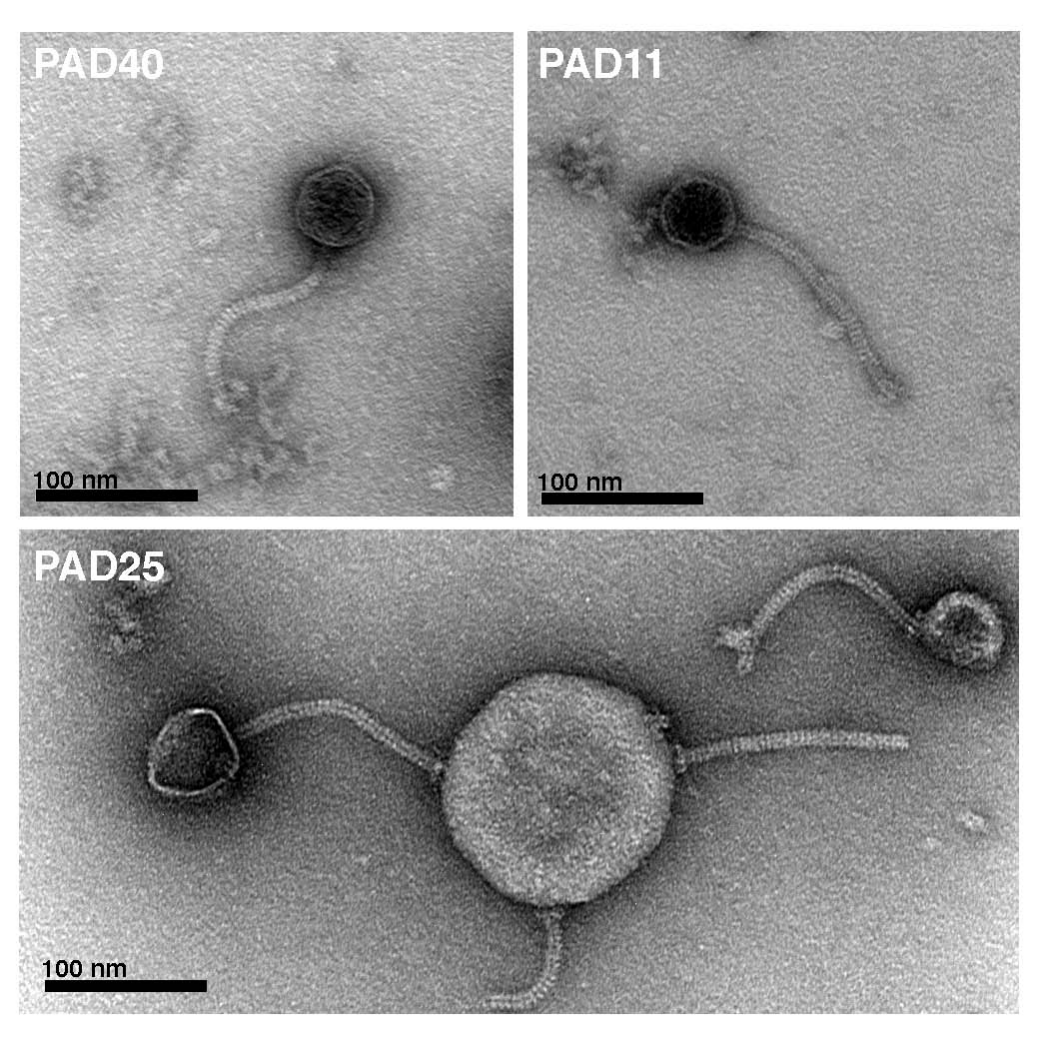
**Electron micrographs of bacteriophages from *P. acnes***. Phages were negatively stained with 0.75% uranyl formate and subjected to transmission electron microscopy. The phages have a head of approximately 55 nm in diameter, loaded with genetic material. Their tails have a size of 150 × 10 nm and are flexible and non-contractile. In the lower micrograph, PAD25 is adhering to bacterial cell debris, and two phages have lost their heads. At the attachment site between the phage and the cell debris, a base plate with attached spikes can be observed. All phages were classified as Siphoviruses based on their morphology.

**Figure 3:**
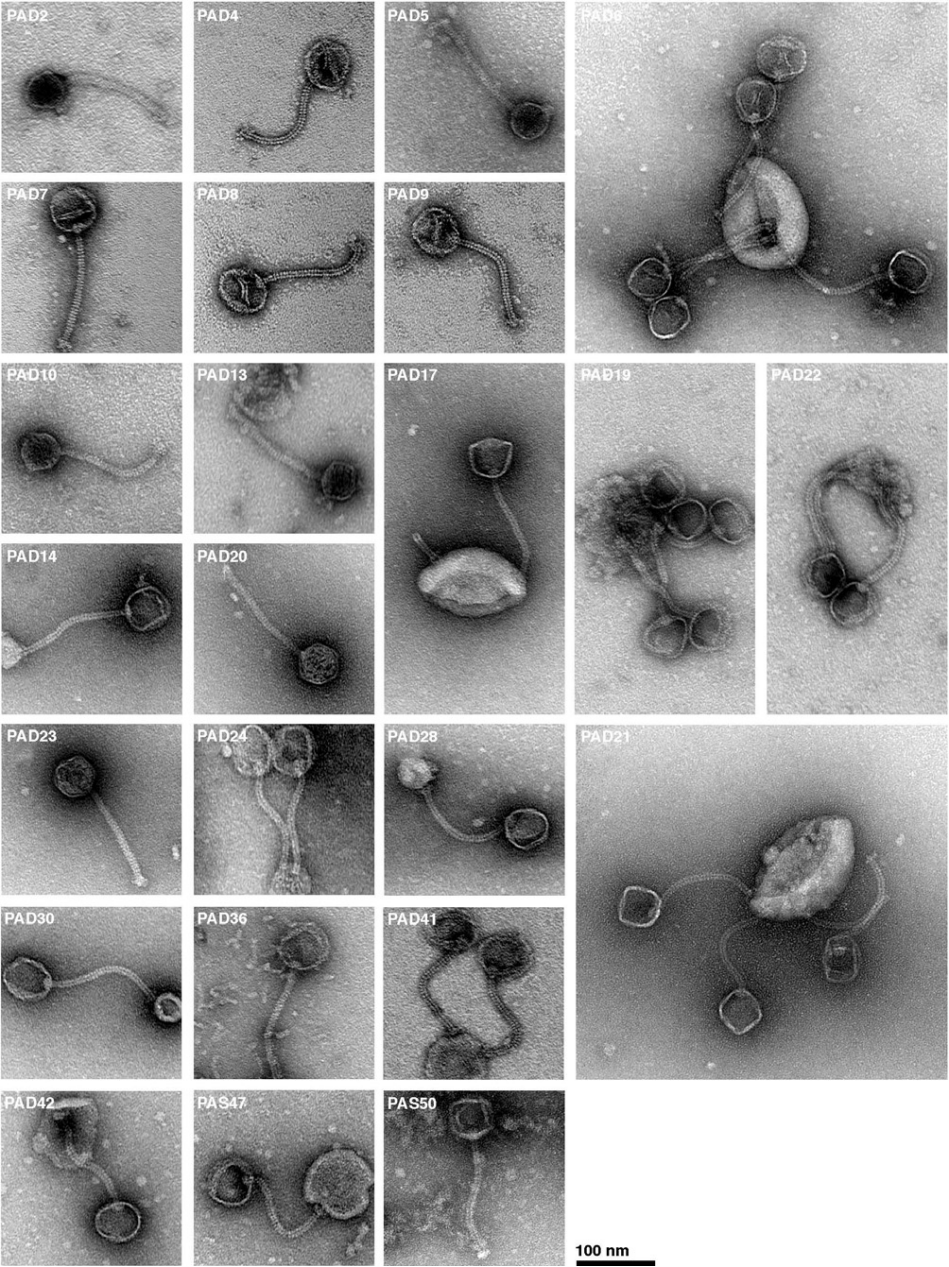
***P. acnes *bacteriophages classified as Siphoviruses**. Phages were negatively stained with 0.75% uranyl formate. All phages were classified as Siphoviruses based on their morphology. No difference in morphology could be observed between the different phages. Several of the phages have empty heads and adhere to bacterial cell debris.

The classification of bacteriophages is mainly based on phage morphology and the nature of the nucleic acid [30]. Though other classification systems such as sequence similarities within genes encoding structural proteins have been proposed, classification based on morphology and the nature of the nucleic acid is still the most accepted system [31]. Forty-nine of the bacteriophages were examined using negative staining and transmission electron microscopy. All examined phages have an icosahedral head of approximately 55 nm in diameter, and a tail composed of 33 segments with a total length of 145–155 nm and a width of 9–10 nm. The tail is non-contractile and appears flexible. Most phages have a visible base plate on the tail with attached spikes (see Figure 2, 3). These morphological attributes warrant classification of the phages as *Siphoviridae*. Thus, this morphology is identical to PA6 [29] and very similar to the *P. acnes *phages studied by Zierdt [32]. Also, the phages are very similar morphologically to Siphoviruses isolated from other propionibacteria [24,25,27]. This classification was further strengthen by amino acid sequence comparison of a part of a putative major head protein with other known phage proteins, using a BLASTp search against GenBank. The best hit was on gp6 from PA6, and the second and third best hit on gp7 from *Mycobacterium *phage Che9d and gp7 from *Mycobacterium *phage Halo. All these phages are classified as *Siphoviridae*.

### Phage specificity

Bacteriophages are generally quite restricted in their host range. There are phages that can infect over bacterial species boundaries, but some phages are species specific and in many cases also specific for certain subgroups (subspecies, serotypes or biotypes, strains) within a species. To determine how specific the isolated phages are, different *P. acnes *isolates were infected with 48 different phages (see additional file 1). Bacterial isolates with inducible phages were generally easy to lyse, with plaque production in nearly 99% (2278/2304) of cases. *P. acnes *isolate AS12 was significantly more difficult to lyse than other isolates with inducible phages, and only 73% (35/48) of the examined phages caused plaque formation. Similarly, phage PAD8 failed to lyse 19% (9/48) of the examined isolates. In *P. acnes *isolates without any inducible phages, we could only observe plaque formation in 30% (144/480) of the cases. We also examined the ability of phages to cause lysis in the recently sequenced *P. acnes *strain KPA171202 (DSM no. 16379) of biotype IB [9]. This strain was lysed only by 40% (19/48) of the phage lysates. The different phages are not specific to certain biotypes, but seem to be less lytic against biotype II in isolates with inducible phages, since 23/26 failures to infect and lyse the bacterial isolates were in isolates of biotype II.

**Figure 4:**
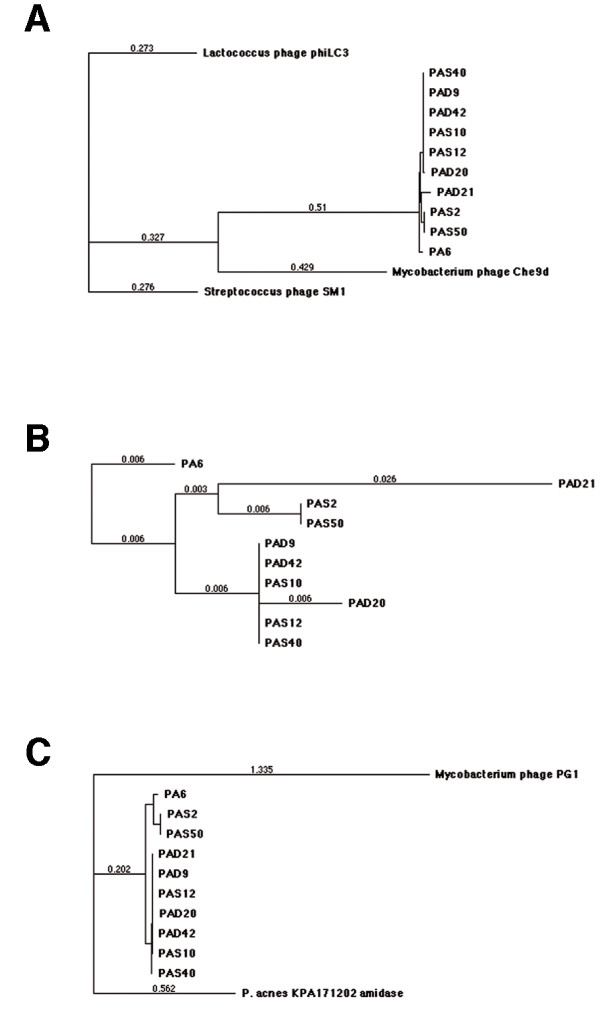
**Phylogenetic trees of phages from *P. acnes***. A gene encoding a putative major head protein and a gene encoding a putative amidase were sequenced in nine *P. acnes *phages and aligned using MacVector ClustalW alignment. Phylogenetic trees were constructed using neighbor joining with best tree mode. The putative major head protein (A) was similar between all *P. acnes *phages examined and showed the highest similarity to *Mycobacterium *phage Che9d gp7, but did also have high similarity to *Lactococcus *phage phiLC3 MHP and *Streptococcus *phage SM1 gp40. If outgroups were removed (B) four separate groups of major head proteins could be observed. One group with PA6, another with PAS2 and PAS50, a third group with PAD21 and a forth group with PAS10, PAS12, PAS40, PAD9, PAD20 and PAD42. The putative amidase (C) showed similar patterning among the phages with phages PAS2 and PAS50 representing one group closely related to PA6, while the other phages PAS10, PAS12, PAS40, PAD9, PAD20, PAD21 and PAD42 formed a second group. The closest known phage protein with similarity to the putative amidase is represented by *Mycobacterium *phage PG1 gp49, but more related is *P. acnes *own amidase.

**Figure 5:**
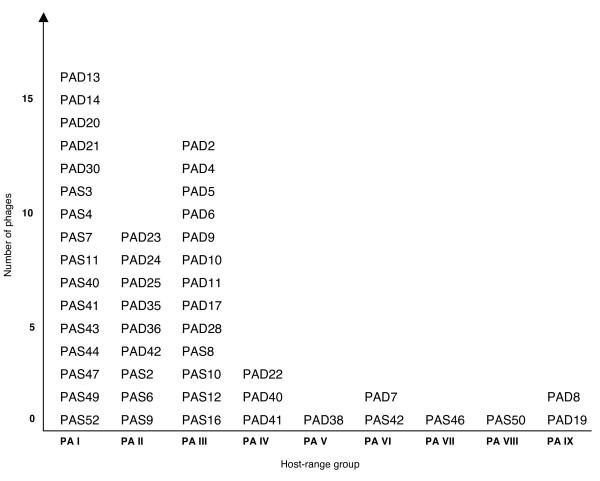
***P. acnes *bacteriophages host-range groups**. The host-range for phages isolated from *P. acnes *was determined by using a bacterial overlay of different *P. acnes *isolates and adding phages. Four bacterial isolates (KPA171202, AD7, AS1 and AS5) were used to divide the phages into different host-range groups. Phages in host-range group PA I could infect and lyse all four isolates, PA II all except for KPA171202, PA III (AD7, AS1), PA IV (AD7), PA V (AD7, AS5), PA VI (PAD7, PAS42), PA VII (AS1, AS5), PA VIII (AS1, AS5, KPA171202) and PA IX could not infect and lyse any of the isolates used.

Since all of the examined phages have identical morphology, protein pattern and in most cases very high similarity in genes encoding the putative major head protein and an amidase (see Figure 4), we examined if phage host-range could be used as a possible tool to differentiate the phages. Based on the host-range analysis we choose *P. acnes *isolates KPA171202, AD7, AS1 and AS5 to differentiate the phages and to divide them into different groups. By using these four isolates, we could divide the phages into 9 separate groups (see Fig 5). Sixteen of the examined phages could infect and lyse all four bacterial isolates (host-range group PA I). There is a tendency that phages isolated from biotype II and from skin can infect and lyse all four bacterial isolates, and are classified as host-range group PA I. Also, the only phages that can lyse the prophage-free isolates AD26 and AD27 (PAD21, PAS7 and PAS11) are classified as belonging to the host-range group PA I. Furthermore, none of nine selected phages (PAS2, PAS10, PAS12, PAS40, PAS50, PAD9, PAD20, PAD21 and PAD42) were able to infect and lyse *P. avidum*, *P. granulosum *or *P. freudenreichii *(data not shown). Our results show that isolates lacking inducible temperate phages are more difficult to lyse using phages, than isolates carrying inducible temperate phages and that the phages are specific to *P. acnes*.

### Phylogenetic analysis of phage genes and proteins

A part of a putative major head gene was sequenced in all isolated phages. The nucleotide sequence was aligned and a phylogenetic tree was reconstructed. The phages could be divided in two distinctly divided groups with the already sequenced phage PA6 forming a third group (see additional file 2). The whole gene encoding a putative major head protein was sequenced in nine of these phages, selected based on their partial sequencing and site of isolation (AD/AS), and the amino acid sequences were aligned and a phylogenetic tree was reconstructed (see Figure 4). The *P. acnes *phage putative major head protein show high similarity to phages isolated from *Mycobacterium*, *Lactococcus *and *Streptococcus*. The already sequenced phage PA6 forms its own group, while PAD9, PAD42, PAS10, PAS12, PAS40 and PAD20 form a large group with similar sequences, even though PAD20 is slightly different. Phages PAS2 and PAS50 form a third group and PAD21 forms a fourth separated group. This pattern is similar to the pattern seen when only using partial sequencing, except that PAD21 now seems to form its own group.

We further sequenced the gene encoding a putative amidase in these nine phages (see Figure 4C). The phage pattern is similar to what could be seen when aligning the major head protein. However, PA6 is more closely related to PAS2 and PAS50 in this protein, and PAD21 do not form its own group, but have high similarity to the large group of phages. The closest phage similarity is to *Mycobacterium *phage PG1 gp49, but more closely related is a chromosomally encoded *P. acnes *amidase.

## Discussion

We show that more than 70% of the investigated *P. acnes *isolates are carriers of inducible phages, which is a significantly higher carriage rate of inducible phages than that reported by Webster *et al *(18%) [20] despite that similar methods were used and isolates were from both skin and deeper infections. This is most likely due to differences in the geographic origin of isolates, and may reflect a difference in *P. acnes *susceptibility to mitomycin C or strain acquired resistance to phages. There was no overall difference in carriage rate of inducible phages between *P. acnes *isolated from skin or deep infections, indicating that carriage of phage not necessary leads to an increased virulence of the host strain. Thus it appears that the phages studied here do not carry virulence factors, which is in concordance with the lack of putative virulence factors on the recently published genome of a *P. acnes *phage [29]. It should be noted that at present, we only have information on the presence or absence of inducible phages. However, future studies may very well reveal differences in the gene composition of phages isolated from skin and deep isolates.

We found that isolates of biotype IB have significantly lower rate of inducible phages as compared to isolates of IA and II. Interestingly, none of the isolates of biotype IB in isolates from deep infections are carriers of inducible phages (0/7), while isolates from skin of biotype IB have a carriage rate of almost 73%. The low carriage rate of inducible phages in AD-isolates typed as IB is further strengthen by the fact that none of these isolates have similarities to the phage major head gene as judged by failure of amplifying this gene in these isolates (data not shown), thus indicating that these isolates do not have any inducible phages and not any prophages with similarities to known *P. acnes *phages. Thereby it seems like either only biotype IB without phages can cause infections, or that biotype IB loses their phages during infection. If this is the case, it is uncertain why this only happens in biotype IB and not in biotype IA and II. The lower carriage rate in biotype IB is neither due to an increased resistance to phage entrance into the bacterial cell, since biotype IB is equally sensitive to phages as the other biotypes, as judged by additional file 1. More research in this specific area is needed to understand this phenomenon.

When we examined the host isolate specificity of the phages, we found that isolate AS12 was significantly more difficult to lyse than the other isolates with inducible phages. However, AS12 is still highly susceptible to PAS12, indicating that AS12 might differ in a protein or receptor essential for most phages to be able to infect and lyse the bacterial cell. Opposite to this, we found that phage PAD8 did not lyse other isolates as efficiently as other phages. This difference could be caused by the phage favouring a lysogenic state, or that the phage receptor binding to the bacterial cell is less efficient compared to other *P. acnes *phages. When we added phages to *P. acnes *isolates without inducible phages, we found that most of these isolates were resistant to phage-mediated lysis. This is not surprising, since these isolates do not have inducible phages and thereby could have a mechanism that makes them resistant against phage infection. The different phages were not specific to certain biotypes. However, most of the failures to infect and cause lysis in the bacterial isolates were in biotype II, possibly indicating that these isolates generally are more resistant to phages, or adapt more easily to them. Grouping of the phages based on host-range did not correlate with biotype, even though there is a tendency that phages from isolates of biotype II are classified as host-range group PA I, thus indicating that phages from isolates of biotype II may have broader infective capacity as compared to biotype IA and IB. The phages also seem to be specific to *P. acnes *isolates, since none of the examined phages were able to infect and lyse *P. avidum*, *P. granulosum *or *P. freudenreichii*. All phages examined were able to infect and lyse their non-induced parental host, thus indicating that the prophages do not confer superinfection immunity as many other known prophages do [33,34]. This is in concordance with the fully sequenced phage PA6 that does not seem to have a repressor-like protein [29]. This may benefit *P. acnes *phage evolution by more efficient gene transfer between prophages and free phages.

The phages examined could be divided into three-four groups using phylogenetic analysis of the gene encoding a putative major head protein and an amidase. This differences in sequence stress the fact that the phages in the different groups very well may have other genetic dissimilarities, providing advantages for the phages. It is also obvious that several phages have identical nucleotide sequences and perhaps should be considered as subspecies to certain groups of *P. acnes *phages. The changes in the gene encoding the major head do not lead to a changed morphology, as seen when comparing PAS50 with the other phage micrographs (see Figure 3). When examining the phage proteins on an SDS-PAGE, all phages have identical protein patterns with four protein bands (17 kDa, 29 kDa, 52 kDa and 54 kDa, data not shown). This indicates that the phages have very similar structural proteins, even though some substitution in amino acids occur, and may reflect an evolutionary pressure to retain the structural proteins.

## Conclusion

We have induced, identified, and characterized 65 temperate *P. acnes *phages, classified as Siphoviruses on a morphological basis. These phages are species specific and do not confer superinfection immunity. These results give new insight into the relation between *P. acnes *and its phages, and contribute to a better understanding of the phage-host interaction.

## Methods

### Bacterial isolates

A total of 92 *P. acnes *isolates were used. These were divided into two groups: 44 isolates from deep infections (AD-isolates, mainly isolated from prosthesis and sternal wires) and 48 isolates from skin from healthy individuals (AS-strain), described in Holmberg *et al*, unpublished. All isolates from healthy individuals and AD-isolates 1–16 and 37 are isolated in Lund, Sweden. AD-isolates 17–32 and 38–48 are isolated in Örebro, Sweden, and AD-isolate 33–36 are isolated in Malmö, Sweden. The *P. acnes *strain KPA171202 (DSM no. 16379), *P. avidum *(DSM no. 4901), *P. granulosum *(DSM no. 20458) and *P. freudenreichii *subsp. *freudenreichii *(DSM no. 20271) were obtained from DSMZ (Deutsche Sammlung von Mikroorganismen und Zellkulturen GmbH, Braunschweig, Germany).

### Isolation of phages

*P. acnes *isolates were plated from frozen stocks on Tryptic Soy Broth (TSB, Bacto, Mt Pritchard, NSW, Australia) with 1.5% Agar (TSBA, Bacto), and incubated for two days at 37°C under anaerobic conditions. Isolates were inoculated in 10 ml prereduced TSB (rTSB) followed by incubation for three days. TSB was prereduced by 24 h incubation under anaerobic conditions. The cultures were diluted 1:9 in 1 ml rTSB and incubation continued for 8 h. Mitomycin C (Calbiochem EMD Biosciences, San Diego, CA, USA) was added to a final concentration of 2 μg/ml, and incubation continued overnight. Cultures were centrifugated (10 min, 1,500 g, Eppendorf Centrifuge 5415R) and sterile filtered (Millex-GP 33 mm 0.2 μm, Millipore, Billerica, MA, USA) to obtain a phage stock. The phage stock was stored at 4°C for 7 days to clear the stock from unstable phages [32]. Phages were spotted onto TSBA plates at different concentrations (1:1 – 1:10,000) after a bacterial overlay of the host *P. acnes *isolate had been prepared. Plates were incubated for two days at 37°C under anaerobic conditions and examined for plaques. When single plaques were observed, these were picked with a sterile scalpel and transferred to SM-buffer (20 mM Tris-HCl pH 7.5, 100 mM NaCl, 60 mM MgSO_4_(7H_2_O), 0.01% gelatine) followed by elution overnight at 4°C. Phages were propagated by spotting phages on a TSBA plate with a bacterial overlay of its host *P. acnes *isolate and incubated three days at 37°C under anaerobic conditions. The overlay containing all plaques were transferred to SM-buffer, eluted overnight at 4°C, followed by sterile filtration of the SM-buffer. This method generally generated a concentration of 10^11^–10^13 ^pfu/ml. Phages were named after the host bacterium with a 'P' before the bacterial isolate name. Isolates were regarded as having inducible phages if infectious phages were induced using the method described above. All bacterial isolates that did not have any inducible phages were screened for prophages using a PCR-based amplification of the major head gene (primer pair MHF/MHR) and using *recA *amplification as a positive control.

### Phage host specificity

Forty-eight *P. acnes *isolates that carried inducible phages and 10 *P. acnes *isolates that did not carry any inducible phages using mitomycin C were plated on TSBA as an overlay assay. To each plate, 5 μl phage stock (10^10 ^pfu/ml) from 48 different phages was added. Plates were incubated for 2 days under anaerobic conditions and examined for plaques. Nine phages (PAS2, PAS10, PAS12, PAS40, PAS50, PAD9, PAD20, PAD21 and PAD42) were also applied to *P. avidum*, *P, granulosum *and *P. freudenreichii *subsp. *freudenreichii *as an overlay assay.

### Electron microscopy

Bacteriophages in a stock concentration of 10^11^–10^13 ^pfu/ml were placed on a carbon coated copper grid and negatively stained (for references see [35]). A 0.75% uranyl formate solution was obtained by dissolving 37.5 mg uranyl formate (BDH Chemicals Ltd., Poole, UK) in 5 ml boiling water, and stabilized with 5 μl 5 M NaOH. Grids were rinsed for 45 sec with 100 μl TBS and blotted off with a filter paper. The sample (5 μl) was added to the grid, left for 45 sec and blotted off with a filter paper. The sample was washed twice with two 100 μl H_2_O drops and blotted off after each wash with a filter paper. The sample was stained for 3 sec with 100 μl 0.75% uranyl formate and then stained for additionally 15–20 sec with 100 μl 0.75% uranyl formate. Samples were observed using a Jeol JEM 1230 transmission electron microscope operated at 60 kV accelerating voltage, and recorded with a Gatan Multiscan 791 CCD camera.

### Phage gene comparison

Phage lysate (10 μl) was boiled for 10 min to release phage DNA from intact phages. A PCR (Eppendorf Mastercycler personal) was run under the following conditions: 95°C 10 min, 35 cycles of 95°C 1 min, annealing temperature 1 min 30 sec and 72°C 1 min 30 sec, ending with 72°C for 10 min. Final concentration in the mixture was 1× buffer, 0.2 mM dNTP mix, 30 mU/μl *Taq *polymerase, 1.5 mM MgCl_2 _and 1 μM each of the primers. Primers used are described in table 2. All samples were run with an annealing temperature of 56.5°C except when using primer pairs PR264/PAR-2 and MHF/MHR where an annealing temperature of 54°C was used. All reagents except for the primers are from Fermentas (Vilnius, Lithuania). PCR products were washed with SpinPrep PCR Clean-up Kit (Novagen, Madison, WI, USA), sequenced using BigDye Terminator v3.1 Cycle Sequencing Kit (Applied Biosystems, Foster City, CA, USA), and analyzed using an ABI 3100 Genetic Analyzer (Applied Biosystems, Foster City, CA, USA). Sequences were aligned using the Clustal W algorithm [36] and a phylogenetic tree was reconstructed using the MacVector v9.5.2 software package (Cary, NC, USA). The final partial major head gene nucleotide sequences correspond to nucleotides 163–484 in *gp6 *from *P. acnes *bacteriophage PA6. The phylogenetic tree was constructed using UPGMA [37] and uncorrected p-values with a bootstrap with 1000 replications. Phylogenetic trees including outgroups with protein comparisons of a putative major head and an amidase were constructed using neighbor joining and poisson-correction with a best tree mode, while phylogenetic trees without outgroups were analysed using uncorrected p-values.

**Table 2:** Primers used

Name	Sequence (5'-3')	Reference
PR264	GCAGGCAGAGTTTGACATCC	[38]
PAR-2	GCTTCCTCATACCACTGGTCATC	[39]
MHF	TCCTGGTTCTATGATTGGTGCG	This study
MHR	CGGAGACCCCTTCGGATACAC	This study
MH1F	CGTTTGTGGATGCTCTTGTCA	This study
MH1R	CCTTCGGATACACCTCAGTAGACA	This study
MH2F	GCTCTTGGTGCTTCGATTGGT	This study
MH2R	GATACCCATCAACACCACCCC	This study
Ami1F	GGTTTGAATGGTGTGAAAGGTC	This study
Ami1R	TTTCGGAACATTATATTTGTCACAC	This study
Ami2F	TATCGAGATTTGCGCGGAT	This study
Ami2R	ACCACGAAACGACTCCGC	This study

### Statistical methods

All statistical tests were calculated using the Chi-Square test. All tests were also run with Fischer's exact test with similar results.

### Nucleotide sequence accession number

All partial sequences of the gene encoding a putative major head were submitted to GenBank. Accession numbers (EU302607–EU302671) are shown in table 1. Whole sequences of the gene encoding a putative major head for phages PAS2, PAS10, PAS12, PAS40, PAS50, PAD9, PAD20, PAD21 and PAD42 have accession numbers EU784673–EU784681 and the gene encoding a putative amidase have accession numbers EU784682–EU784690.

## Authors' contributions

RL participated in the design of the study and performed the isolation of phages, genetic and protein based characterization of phages, host specificity observations and drafted the manuscript. Electron microscopically examinations were done by MM. AH performed the initial characterization of the *P. acnes *strains. MR assisted in statistical analysis and revision of the manuscript. MC designed the study and revised the manuscript. All authors read and approved the final manuscript.

## Supplementary Material

Additional file 1 **Host range analysis**. *Propionibacterium acnes *isolates were plated on TSBA as an overlay assay. Phages isolated from these strains were added to each *P. acnes *isolate and incubated for two days at anaerobic conditions and were analyzed for plaques. A plus (+) indicates that the bacterial isolate was lysed, and the number of + indicates the quality and extent of the lysis in the bacteria. A minus (-) indicates that no plaques were observed. *P. acnes *strain KPA171202 is the recently sequenced *P. acnes *strain. Bacterial isolates AD2-AS52 have inducible phages while bacterial isolates AD1-KPA171202 do not have inducible phages. Click here for file

Additional file 2 **Phylogenetic tree of *P. acnes *phages based on partial sequencing on a gene encoding a putative major head protein**. A part of the gene encoding the putative structural protein major head protein was amplified and sequenced. Obtained nucleotide sequences were compared using MacVector ClustalW Alignment and a phylogenetic tree was constructed using UPGMA and uncorrected p-values with 1000 replications for bootstrap. The phages were divided into two distinct groups, with the recently sequenced phage PA6 forming a third group. Click here for file
