# Supplementary figures and images for: Inducible Siphoviruses in superficial and deep tissue isolates of Propionibacterium acnes
Source: BMC Microbiol. 2008 Aug 15;8:139. doi: 10.1186/1471-2180-8-139 (PMC2533672; doi:10.1186/1471-2180-8-139)

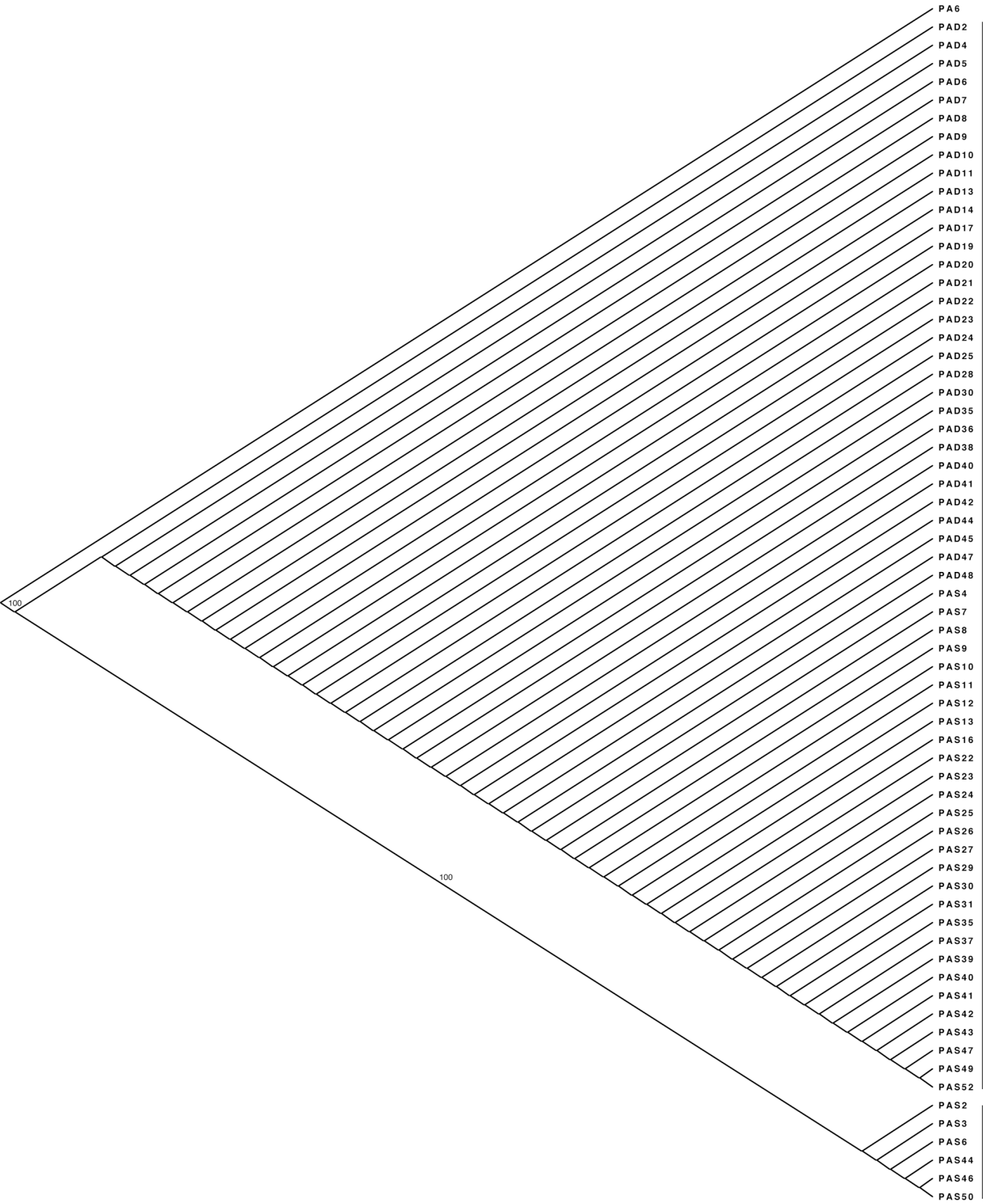

Supplement: Additional file 2 — Phylogenetic tree of P. acnes phages based on partial sequencing on a gene encoding a putative major head protein. A part of the gene encoding the putative structural protein major head protein was amplified and sequenced. Obtained nucleotide sequences were compared using MacVector ClustalW Alignment and a phylogenetic tree was constructed using UPGMA and uncorrected p-values with 1000 replications for bootstrap. The phages were divided into two distinct groups, with the recently sequenced phage PA6 forming a third group. [file 1471-2180-8-139-S2.pdf]
